# The impact of the time of day on muscle and metabolic responses to resistance exercise in healthy adults: A randomised controlled trial

**DOI:** 10.1113/EP093020

**Published:** 2025-11-13

**Authors:** Anas Dighriri, Hannah Lithgow, Brendan Gabriel, Mazin Altuwrqi, Emma Dunning, Lynsey Johnston, James G. Boyle, Greig Logan, Stuart R. Gray

**Affiliations:** ^1^ School of Cardiovascular & Metabolic Health University of Glasgow Glasgow UK; ^2^ School of School of Energy, Geoscience, Infrastructure and Society Heriot‐Watt University Edinburgh UK; ^3^ School of Medicine, Medical Sciences and Nutrition University of Aberdeen Aberdeen UK; ^4^ Institute of Sports Science and Innovation Lithuanian Sports University Kaunas Lithuania

**Keywords:** circadian rhythm, exercise timing, metabolic responses, resistance exercise

## Abstract

Resistance exercise provides numerous health benefits, including improved glucose control and enhanced muscular strength. However, it remains unclear whether the time of day resistance exercise is performed affects these benefits. The objective of this work was to determine the effect of time of day on muscle and metabolic responses to resistance exercise training in young healthy adults. The study included 36 participants (30 ± 7 years old; and 28 ± 4 kg/m^2^) who were randomised into control, morning (06.00–10.00 h) or evening (16.00–20.00 h) groups. Exercise groups performed eight resistance exercises, 3 times per week, for 6 weeks, at their allocated time. At baseline and post‐intervention, insulin sensitivity, flash glucose monitor data, muscle strength and vastus lateralis muscle thickness were measured. Over the 6‐week intervention, there were significant main effects of time on insulin sensitivity (*P *< 0.001), muscle thickness (*P* = 0.008) and knee extensor maximal torque (*P *< 0.001), indicating improvements with resistance exercise training. However, there were no significant time × group interactions for any outcome measures (insulin sensitivity *P* = 0.206, muscle thickness *P* = 0.279, knee extensor torque *P* = 0.151), demonstrating that exercise timing did not differentially affect training adaptations. Both exercise groups showed similar improvements compared to controls, regardless of whether training occurred in the morning or evening. No significant effects were observed for flash glucose monitor data. This study highlights the benefits of resistance exercise and demonstrates that timing has little influence on these effects. Promotion of resistance exercise at convenient times is recommended. This study was registered at ClinicalTrials: ClinicalTrials.gov ID: NCT05321914.

## Abstract (highlights)

- **What is the central question of this study?**
  What is the effect of time of day on muscle and metabolic responses to resistance exercise training in young healthy adults?
- **What is the main finding and its importance?**
  In healthy adults, 6 weeks of resistance exercise resulted in an improvement in muscle strength and muscle mass, with no clear effect of the time of day at which exercise was performed. In healthy adults, 6 weeks of resistance exercise resulted in an improvement in insulin sensitivity, with no clear effect of the time of day at which exercise was performed.

## 1 INTRODUCTION

Chronobiology is the study of the human internal body clock, which regulates sleep cycles, body temperature, feeding and hormone secretion (Brown et al., 2002; Buhr et al., 2010). At a molecular level, circadian rhythms are controlled by transcriptional activators CLOCK and BMAL1 as well as their target genes period (PER) and cryptochrome (CRY) (Gerhart‐Hines & Lazar, 2015; Robinson & Reddy, 2014). This is approximately a 24 h transcription–translation feedback loop, which drives the entire body's metabolism (Gabriel & Zierath, 2019). Changes in the circadian rhythm can increase the risk of chronic diseases such as hypertension, diabetes and cancer (Forman et al., 2010; Schernhammer et al., 2001). For example, metabolic disease has been associated with a disruption of mammals' circadian clocks (Panda, 2016). On top of this, variations in glucose tolerance and insulin action throughout the day highlight that glucose metabolism is influenced by circadian rhythms (Gagliardino et al., 1984; Van Cauter et al., 1997). It is possible that strategies to benefit metabolic health may interact with these circadian rhythms and alter their effects.

One such strategy is exercise, which is an integral component of diabetes management, with both aerobic and resistance exercise recommended (Diabetes Canada Clinical Practice Guidelines Expert et al., 2018). Both forms of exercise have broad health benefits (Diabetes Canada Clinical Practice Guidelines Expert et al., 2018; Sigal et al., 2004; Yardley et al., 2013), with less research on resistance compared to aerobic exercise. Resistance exercise has the benefit of increasing muscle mass and strength, which are important for maintenance of physical function, blood pressure and glucose control and for morbidity/mortality risk (Celis‐Morales et al., 2018; Colberg et al., 2010; Kirwan et al., 2017). Performance responses to exercise vary according to time of day; for example, power and strength peak in the early evening hours (Drust et al., 2005), and there has been a suggestion that some of the benefits of exercise may be modulated by the time of day at which it is performed. For example, it has been hypothesised that evening exercise may be more effective than morning exercise for improving insulin sensitivity due to circadian variations in glucose metabolism (Heden & Kanaley, 2019). Building on this, in people with type 1 diabetes, blood glucose responses to exercise vary depending on the time of day at which resistance exercise is performed (Toghi‐Eshghi & Yardley, 2019), but data from people without diabetes are limited (Tanaka et al., 2021). There are also limited data on whether increases in muscle strength and mass vary depending on when resistance exercise is performed, although data tentatively indicate no such effect is present (Grgic et al., 2019), and further work is needed to confirm these findings.

The aim of the current study, therefore, is to determine the effect of time of day on the muscle and metabolic responses to resistance exercise in young healthy adults.

## 2 METHODS

The current study was registered on 11 April 2022, with ClinicalTrials.gov, ID: NCT05321914.

### 2.1 Ethical approval

The study was approved by the ethical review committee of the College of Medical Veterinary and Life Sciences at the University of Glasgow (approval number: 200210068). All participants provided written informed consent prior to participation. The study adhered to the guidelines of the *Declaration of Helsinki*.

### 2.2 Participants

Participants were recruited through flyers and social media posts. Inclusion criteria were: age between 18 and 45 years old, body mass index (BMI) >23.0 to 40 kg/m^2^ and self‐reported participation in less than 1 h of structured exercise training per week. Exclusion criteria included: having undergone surgery for weight loss and a prior history of heart, lung, cancer, kidney, endocrine or liver disease.

### 2.3 Study design

**FIGURE 1:**
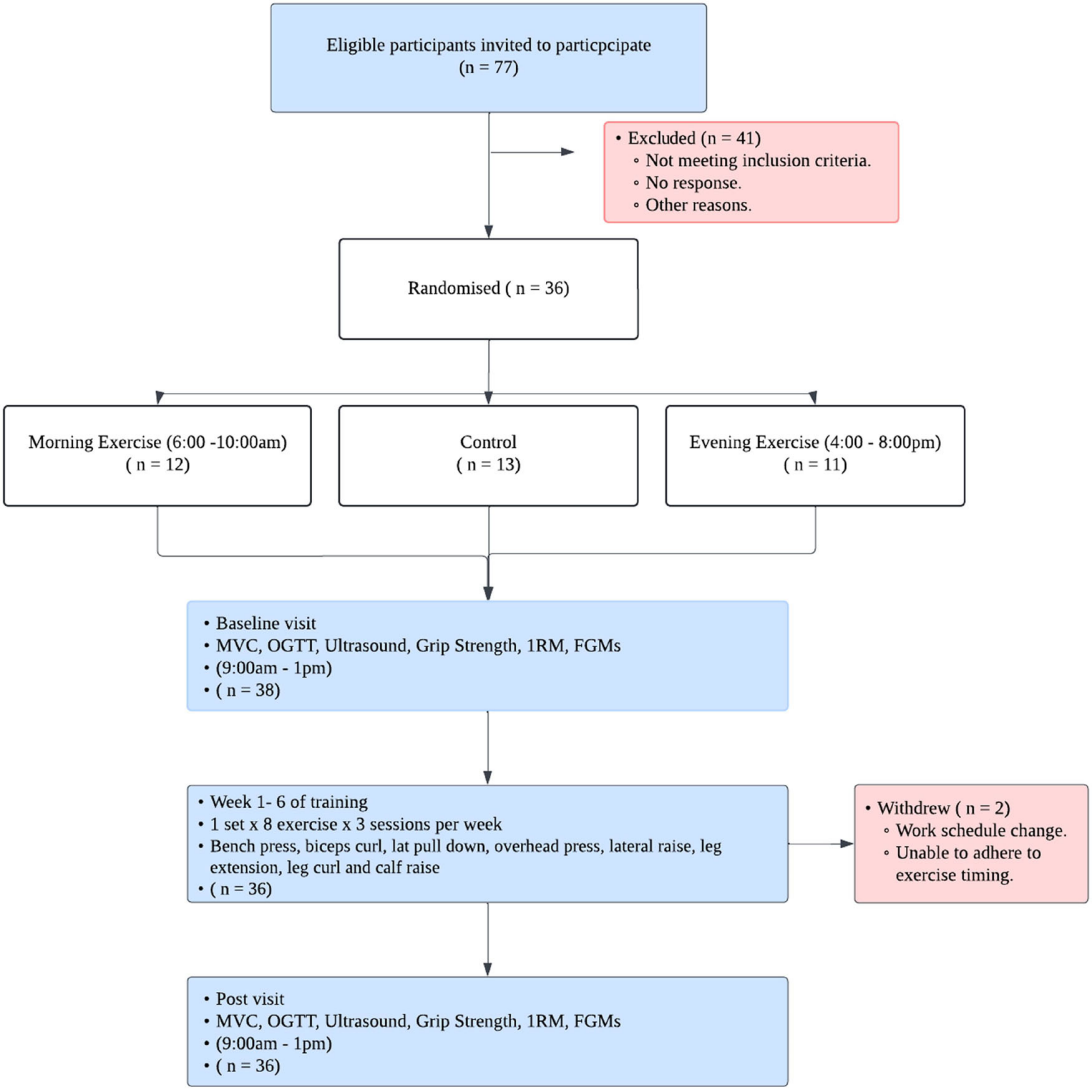
Flow diagram of participant recruitment, randomisation and follow‐up.

Following baseline assessment of Munich Chronotype Questionnaire (MCTQ), insulin sensitivity via oral glucose tolerance test (OGTT), body composition, vastus lateralis muscle thickness, knee extensor maximal torque and grip strength and one‐repetition maximum (1RM) were measured for resistance exercises. Participants were then randomised to either a control, exercise in the morning (06.00–10.00 h) or exercise in the evening (16.00–22.00 h) group for the 6‐week intervention period. The allocation sequence was generated by an independent researcher, and participants' assignments were contained within a sealed opaque envelope. Study investigators were blinded to the initial allocation sequence but not the final group assignment. Following the baseline assessment, interstitial glucose levels were measured via a blinded flash glucose monitor (FGMs) for 14 days. This included the 7 days prior to beginning the intervention and the first 7 days of the intervention. Following the 6‐week intervention, a further assessment of insulin sensitivity, body composition, vastus lateralis muscle thickness, knee extensor maximal torque, grip strength and 1RM of training exercises was made at least 3 days after the final exercise session. Interstitial glucose levels were measured for the last 7 days of the intervention and 7 days after the end of the intervention. All participants were instructed to maintain their normal dietary habits throughout the study and to repeat the same dietary intake before each testing visit. Participants were asked to fast overnight prior to all study visits and only consume water before coming to the laboratory. Insulin sensitivity was measured beginning around 09.00 h, whilst the strength measurements were made between 12.00 and 13.00 h to ensure they were equidistant between the two training times. The study flow of participants, including recruitment, randomisation and follow‐up, is illustrated in Figure 1.

### 2.4 Equipment and materials

#### 2.4.1 Body composition assessment

Bioelectrical impedance analysis was performed using a Tanita body composition analyser (Tanita Corporation, Tokyo, Japan).

#### 2.4.2 Muscle thickness measurement

Vastus lateralis muscle thickness was assessed using portable brightness mode (B‐Mode) ultrasound imaging (Echo blaster 128 Ext; Telemed Ltd, Vilnius, Lithuania). Using a 7.5 MHz linear array transducer, the B‐Mode scan probe was placed on the lateral surface of the thigh at 70% of the distance between the lateral condyle of the femur and the greater trochanter as previously described (Ismail et al., 2019).

#### 2.4.3 Muscle strength assessment

Knee extensor maximal voluntary contraction (MVC) was measured using an isometric dynamometer with force recorded via a load cell (Biometrics Ltd, Newport, UK) with participants positioned at a knee angle of 90°. A minimum of three contractions (up to 5–10 s, with 3 min rest between contractions) were performed, and the highest value was used for analysis. Grip strength was assessed using a hand grip dynamometer.

#### 2.4.4 Insulin sensitivity (oral glucose tolerance test, OGTT)

A cannula was inserted into an antecubital vein, and a baseline blood sample was collected. Participants consumed 75 g of glucose made up to 300 mL with water, and further blood samples were collected after 30, 60, 90 and 120 min.

#### 2.4.5 Continuous glucose monitoring

Flash glucose monitoring was performed using FreeStyle Libre Pro sensors (Abbott Diabetes Care, Alameda, CA, USA) applied to the posterior aspect of the upper arm for 14‐day monitoring periods.

#### 2.4.6 Biochemical analysis

Blood glucose was measured on a C311 analyser and insulin on an e411 analyser (Roche Diagnostics, Burgess Hill, UK), with all assays calibrated and quality control using the manufacturer's reagents.

### 2.5 Interventions

#### 2.5.1 Control

The control group were asked to maintain their habitual physical activity levels.

#### 2.5.2 Exercise

Exercise consisted of 1 set × 8 exercises × 3 sessions per week for 6 weeks. The load was 80% of 1RM with each set performed to volitional failure. All sessions of training were supervised by a researcher, and the following exercises were performed: bench press, biceps curl, lat‐pull down, overhead press, lateral raise, leg extension, leg curl and calf raise. The Smith machine was used for bench press, overhead press and calf raises, whilst a pully machine was used for biceps curl, lat‐pull down, leg extension and leg curl, and dumbbells were used for lateral raises. Training volume was quantified for each exercise as weight × number of repetitions.

### 2.6 Outcome measurements

#### 2.6.1 Munich Chronotype Questionnaire

A self‐reported sleep form was given to all participants during the baseline and post‐intervention, to evaluate their sleep and chronotype (Ghotbi et al., 2020).

#### 2.6.2 Insulin sensitivity (oral glucose tolerance test, OGTT)

A cannula was inserted into an antecubital vein and a baseline blood sample was collected. Participants consumed 75 g of glucose made up to 300 mL with water, and further blood samples were collected after 30, 60, 90 and 120 min. Blood samples were analysed for glucose and insulin levels in our clinical biochemistry laboratory.

#### 2.6.3 Body composition

Bioelectrical impedance analysis (BIA) was used to measure lean and fat mass using a Tanita body composition analyser (Tanita Corporation).

#### 2.6.4 Vastus lateralis muscle thickness

Participants were asked to lie on an examination couch, and an ultrasound device was used to measure the thickness of the vastus lateralis.

#### 2.6.5 Knee extensor maximal torque

Participants were strapped in a chair with their legs at a 90‐degree angle, and a strap was placed around the right ankle, which was connected to a force transducer. Participants were asked to contract maximally for 5–10 s with the leg fixed in position, and force was recorded.

#### 2.6.6 Grip strength

Participants were asked to perform three maximal contractions, on each hand, with the hand grip dynamometer.

#### 2.6.7 One‐repetition maximum

All participants were asked to do 1RM for eight exercises: bench press, biceps curl, lat‐pull down, overhead press, lateral raise, leg extension, leg curl and calf raise. For upper muscle exercises, bench press, biceps curl, lat‐pull down, overhead press and lateral raises were combined as ‘upper body exercises 1RM’. For lower muscle exercises, leg extension, leg curl and calf raises were combined as ‘lower body exercises 1RM’ for data analysis.

#### 2.6.8 Flash glucose monitoring

All participants were fitted, in the back of the upper arm, with blinded flash glucose monitoring (FGM) sensors for 14 days.

### 2.7 Data and statistical analysis

The glucose and insulin area under the curve were calculated during the OGTT using the trapezoid rule. On top of this, glucose and insulin data were also used to estimate insulin sensitivity via the Matsuda Index (Matsuda & DeFronzo, 1999) as follows:

$$\begin{array}{ccc} & & \mathrm{Insulin}\;\mathrm{sensitivity}\\ & & \;=\frac{10000}{\sqrt{\left(\mathrm{FPG}\times \mathrm{FPI}\right)\times \left(\mathrm{mean}\;\mathrm{OGTT}\;\mathrm{glucose}\times \mathrm{mean}\;\mathrm{OGTT}\;\mathrm{insulin}\right)}} \end{array}$$

Using flash glucose monitoring (FGM) data, mean and standard deviation of glucose were calculated using EasyGV (Hill et al., 2011) over four time periods.

1. The 6 h period post‐exercise for both morning and evening exercise groups (week 1 and 6) is named as ‘6 h post‐exercise’ for data presentation.
2. The 24 h period on days of exercise for all three groups, with an average of all 24 h periods used in the control group (week 1 and 6), named as ‘24 h exercise’ for data presentation.
3. The 24 h period on days following exercise for all three groups, with an average of all 24 h periods used in the control group (week 1 and 6), named as ‘24 h post‐exercise’ for data presentation.
4. The 24 h period averaged over all 7 days for all three groups (week 0 and 7), named as ‘24 h pre‐post’ for data presentation.

All data were tested for normality and skewness using Shapiro–Wilk tests and visual inspection of Q–Q plots. Skewness and kurtosis were evaluated to confirm normal distribution assumptions before selecting the appropriate test. A two‐way (group × time point) repeated measures analysis of variance (ANOVA) was conducted using SPSS version 28.0 (IBM Corp., Armonk, NY, USA) to compare data pre‐ versus post‐intervention, with a *P*‐value of <0.05 being used to determine statistical significance. Data are presented as means ± standard deviation (SD). The current study is an exploratory study, and no formal sample size calculation has been carried out. However, the aim was to recruit 36 participants (12 per group) to allow us to detect a 1.15 SD difference in outcomes. Statistical significance was set at a *P *< 0.05.

## 3 RESULTS

### 3.1 Participant recruitment and characteristics

Out of 77 individuals screened for eligibility, 36 healthy adults (19 males, 17 females: age 30 ± 7 years old) met the inclusion criteria and were randomised into three groups: morning exercise (*n* = 12), control (*n* = 13) and evening exercise (*n* = 11) (Figure 1). Additionally, all 36 participants completed the MCTQ at both times. Sleep corrected mid‐sleep on free days (MSFsc), which is an indicator of chronotype, was analysed during baseline (5:32 ± 2:00) and during the post‐visit (5:39 ± 1:57). Table 1 presents an overview of the participant baseline characteristics. All participants showed 100% adherence to exercise sessions.

**TABLE 1:** Baseline characteristics of the participants.

Variable	Morning group (*n* = 12)	Evening group (*n* = 11)	Control (*n* = 13)	*P*
Male/Female	3/9	8/3	8/5	
Age (years)	30 ± 7	30 ± 7	30 ± 7	0.711
Height (cm)	169 ± 8	169 ± 8	169 ± 8	0.802
Weight (kg)	80 ± 15	82 ± 14	80 ± 14	0.691
BMI (kg/m^2^)	28 ± 3	28 ± 3	28 ± 3	0.858
MCTQ (MSFsc, h: min)	(5:39 ± 1:54)	(5:38 ± 2:08)	(5:42 ± 1:38)	0.985

Data are means ± SD. BMI, body mass index; MCTQ, Munich Chronotype Questionnaire; MSFsc, sleep corrected mid‐sleep on free days.

### 3.2 Muscle and metabolic data

Muscle and metabolic data are presented in Table 2. A two‐way repeated measures ANOVA revealed a significant effect of time (*P *< 0.001) with insulin sensitivity increasing over the intervention period, no group effect (*P* = 0.815) and no time×group interaction (*P* = 0.206) for the insulin sensitivity index. Additionally, analysis of area under the curve (AUC) revealed significant effects of time for both glucose AUC (*P* = 0.021) and insulin AUC (*P* = 0.047), with both measures decreasing over the intervention period, but no group (glucose *P* = 0.901; insulin *P* = 0.120) or time × group interaction effects (glucose *P* = 0.519; insulin *P* = 0.381) were observed for either measure. Furthermore, no time, group or interaction effects were seen for body fat percentage (time: *P* = 0.456; group: *P* = 0.426; interaction: *P* = 0.245) or fat mass (time: *P* = 0.272; group: *P* = 0.769; interaction: *P* = 0.950). Analysis of vastus lateralis muscle thickness data showed an effect of time (*P* = 0.008) with muscle thickness increasing over the intervention period, but no group effect (*P* = 0.737) or time × group interactions (*P* = 0.279). Analysis of knee extensor maximal torque revealed an effect of time (*P *< 0.001) with torque increasing over the intervention period, but no group effect (*P* = 0.660) and no time × group interaction (*P* = 0.151). Analysis of grip strength showed no time (*P* = 0.195), group (*P* = 0.440) or interaction effects (*P* = 0.061). Lower body 1RM showed no time (*P* = 0.618), group (*P* = 0.457) or interaction effects (*P* = 0.997). Upper body 1RM revealed a significant effect of time (*P *< 0.001) with 1RM increasing over the intervention period, but no group effect (*P* = 0.081) and no time × group interactions (*P* = 0.107). Training volume analysis showed no significant differences in exercise loads between groups across all exercises (*P*‐values ranged from 0.053 to 0.997; Table 3).

**TABLE 2:** Muscle and metabolic outcomes at baseline and post‐intervention.

	Control (*n* = 13)		AM (*n* = 12)		PM (*n* = 11)				
	Pre	Post	Pre	Post	Pre	Post	*P* (time)	*P* (group)	*P* (time × group)
BMI (kg/m^2^)	28.66 ± 3.57	27.15 ± 3.99	27.91 ± 4.54	27.60 ± 4.25	28.06 ± 2.24	27.79 ± 2.36	< 0.001	0.993	0.307
Fat (%)	28.52 ± 6.55	26.45 ± 7.51	30.17 ± 9.16	32.86 ± 7.93	27.09 ± 6.07	29.02 ± 6.78	0.456	0.426	0.245
Fat mass (kg)	23.95 ± 8.06	26.38 ± 14.31	23.97 ± 11.05	26.43 ± 10.89	21.56 ± 4.34	23.19 ± 5.51	0.272	0.769	0.950
Matsuda Index (mg·L^2^ mmol^−1^ mU^−1^ min^−1^)	36.60 ± 28.21	37.30 ± 27.58	35.99 ± 21.62	43.54 ± 24.63	42.97 ± 25.31	49.79 ± 28.73	<0.001	0.815	0.206
Glucose AUC (mmol/L)	5.05 ± 0.61	4.96 ± 0.63	5.19 ± 0.31	4.77 ± 0.30	5.40 ± 0.33	5.06 ± 0.27	0.021	0.901	0.519
Insulin AUC (mIU/L)	67.59 ± 13.60	67.77 ± 17.93	51.29 ± 10.84	36.28 ± 7.80	51.30 ± 6.59	36.58 ± 3.71	0.047	0.120	0.381
Knee extensor maximal torque (N m)	186.46 ± 64.51	188.29 ± 65.60	182.07 ± 26.58	187.61 ± 28.49	195.73 ± 33.68	201 ± 33.34	<0.001	0.660	0.151
Vastus lateralis muscle thickness (mm)	22.20 ± 4.38	22.61 ± 1.60	22.49 ± 3.03	23.60 ± 2.80	23.25 ± 3.73	24.41 ± 3.18	0.008	0.737	0.279
Grip strength (kg)	40.15 ± 11.69	39.62 ± 11.68	35.75 ± 6.18	37.00 ± 6.35	37.55 ± 6.15	39.27 ± 4.74	0.195	0.440	0.061
Upper body exercises 1RM (kg)	114.40 ± 29.72	115.20 ± 32.06	118.00 ± 27.86	129.50 ± 24.20	139.86 ± 19.29	155.29 ± 24.32	<0.001	0.081	0.107
Lower body exercises 1RM (kg)	107.44 ± 29.21	108.96 ± 28.89	118.54 ± 25.57	121.37 ± 18.64	116.43 ± 16.49	118.23 ± 25.37	0.618	0.457	0.997

Values are presented as mean ± standard deviation. Values are time‐averaged and derived using the trapezoid rule. 1RM, one repetition maximum; AM, morning exercise group, AUC, area under curve; BMI, body mass index; ISI, Insulin Sensitivity Index; MVC, maximal voluntary contraction; PM, evening exercise group.

**TABLE 3:** Training volume completed during the 6‐week resistance exercise intervention.

Exercise	Morning group (*n* = 12)	Evening group (*n* = 11)	*P*
Bench press (kg)	138 ± 102.5	203.7 ± 69.1	0.335
Lat‐pulldown (kg)	128.4 ± 30.4	149.4 ± 16.1	0.110
Overhead press (kg)	78.5 ± 66.1	114.9 ± 40.8	0.053
Leg curl (kg)	217.8 ± 55.8	256.3 ± 99.8	0.239
Biceps curl (kg)	111.3 ± 51.5	181.3 ± 34.1	0.454
Lateral raises (kg)	45.3 ± 24.7	55.6 ± 11.2	0.132
Leg extension (kg)	189.2 ± 70.5	190.7 ± 44.8	0.197
Calf raises (kg)	366.2 ± 218.4	361.9 ± 191.2	0.997

Data represent training volume (weight × repetitions to failure) as an average of all 18 training sessions, with data presented as mean ± standard deviation. Each exercise was performed as one set to volitional failure, three times per week.

### 3.3 Flash glucose monitoring

**FIGURE 2:**
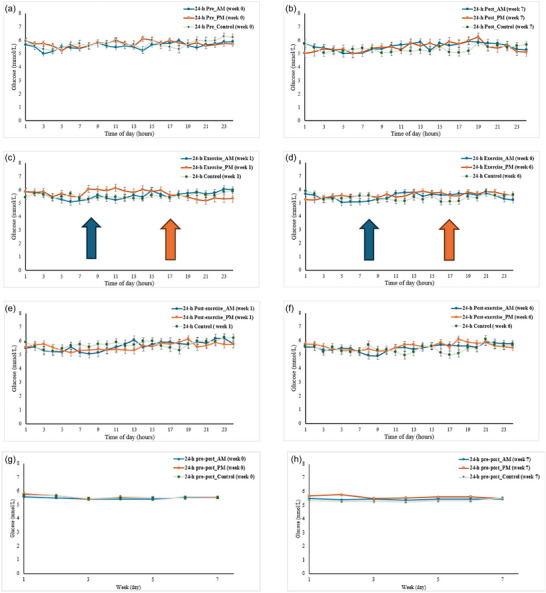
Flash glucose monitoring (FGM) based glucose levels in response to the intervention. (a, b) Comparison of 24‐h pre–post mean glucose levels from FGMs during the first (week 0) and last (week 7) weeks of the intervention. Data are presented for the AM exercise group (blue), PM exercise group (orange), and control group (grey). (c, d). Comparison of mean 24 h exercise glucose levels from FGMs during the first (week 1) and last (week 6) weeks of the intervention. Data are presented for the AM exercise group (blue), PM exercise group (orange), and control group (grey). Arrows indicate exercise timing (AM: 06.00–10.00 h, PM: 16.00–22.00 h). (e, f). Comparison of mean 24 h post‐exercise glucose levels from FGMs on rest days during the first (week 1) and last (week 6) weeks of the intervention. Data are presented for the AM exercise group (blue), PM exercise group (orange). (g, h) comparison of 24 h pre–post exercise glucose levels from FGMs during the first (week 0) and last (week 7) weeks of the intervention.

Flash glucose monitoring analysis revealed no significant main effects of time or time × group interactions for mean glucose or glucose variability (SD) across all measurement periods: 6 h post‐exercise (Figure 2a,b), 24 h exercise days (Figure 2c,d), 24 h post‐exercise days (Figure 2e,f), and 24 h pre–post comparison (Figure 2g,h) (see Table 4 for exact *P*‐values). The exception was a significant main effect of time for glucose SD during 24‐h post‐exercise days (*P* = 0.041) with variability decreasing over the intervention period, though no group effect (*P* = 0.738) or time × group interactions (*P* = 0.701) were observed. Additional glycaemic variability metrics, MAGA, LI, ADDR, J‐INDEX, LBGI & HBGI, CONGA, GRADE and MAG, were analysed and showed no significant effects (data not shown).

**TABLE 4:** Summary of flash glucose monitoring data.

	Control (*n* = 13)		AM (*n* = 12)		PM (*n* = 11)				
	Pre	Post	Pre	Post	Pre	Post	*P* (time)	*P* (group)	*P* (time × group)
Mean 6 h post‐exercise glucose			5.58 ± 0.57	5.42 ± 0.53	5.68 ± 0.36	5.79 ± 0.66	0.721	0.479	0.554
SD 6 h post‐exercise glucose			0.69 ± 0.17	0.60 ± 0.15	0.71 ± 0.16	0.68 ± 0.21	0.495	0.284	0.942
Mean 24 h exercise days glucose	5.53 ± 0.48	5.41 ± 0.39	5.42 ± 0.45	5.45 ± 0.53	5.42 ± 0.28	5.65 ± 0.43	0.372	0.817	0.140
SD 24 h exercise days glucose	0.82 ± 0.35	0.85 ± 0.10	0.77 ± 0.15	0.70 ± 0.14	0.68 ± 0.07	0.70 ± 0.13	0.805	0.430	0.238
Mean 24 h post‐exercise days glucose	5.53 ± 0.48	5.41 ± 0.39	5.58 ± 0.48	5.54 ± 0.71	5.61 ± 0.39	5.66 ± 0.59	0.376	0.574	0.868
SD 24 h post‐exercise glucose	0.82 ± 0.35	0.85 ± 0.10	0.74 ± 0.19	0.68 ± 0.22	0.71 ± 0.12	0.65 ± 0.12	0.041	0.738	0.701
Mean 24 h pre‐post glucose	5.58 ± 0.53	5.41 ± 0.40	5.51 ± 0.48	5.42 ± 0.62	5.65 ± 0.64	5.47 ± 0.53	0.118	0.919	0.647
SD 24 h pre‐post glucose	0.85 ± 0.21	0.80 ± 0.15	0.78 ± 0.17	0.69 ± 0.11	0.73 ± 0.08	0.74 ± 0.14	0.251	0.468	0.807

Mean glucose levels and standard deviation (SD) at 6 h post‐exercise, 24 h exercise, 24 h post‐exercise, 24 h pre–post.

## 4 DISCUSSION

This study aimed to investigate the impact of the timing of resistance exercise on muscle and metabolic responses in young healthy adults. Our findings of effects of time likely reflect the benefits of 6 weeks of resistance exercise on muscle strength, muscle mass and insulin sensitivity with no differences in responses between the groups. These results suggest that the benefits of resistance exercise may be achieved regardless of the time of day for healthy young adults and this should be the focus of public health strategies.

The current study showed an effect of time on insulin sensitivity, with no differences between groups, likely reflecting the established benefits of exercise. Indeed, this improvement in insulin sensitivity with 6 weeks of resistance exercise in healthy young people confirms the findings of Ismail et al. (2019), but has a stronger study design with the inclusion of a control group. This is the first study, to our knowledge, that has compared the effects of the time of day at which resistance exercise is performed on insulin sensitivity, finding no effect, with no difference between the morning and evening groups. This goes against the previous hypothesis put forward that exercise in the evening would be more effective in improving insulin sensitivity (Heden & Kanaley, 2019). On top of this, the current study utilised FGM to monitor glucose responses and found no effect of exercise or time of day on either mean glucose or glucose variability. These findings are in conflict with previous studies investigating interactions between exercise and time of day on glucose responses. For example, Savikj et al. (2019) reported that over a 2‐week intervention period that afternoon high‐intensity interval training is more effective in lowering glucose levels compared to morning training in people with type 2 diabetes. On top of this, Toghi‐Eshghi & Yardley (2019) reported that, in people with type 1 diabetes, after an acute bout of morning (fasting) resistance exercise, there was an increase in blood glucose, whilst it declined during afternoon resistance exercise, with higher glucose variability and more frequent hyperglycaemic events after morning exercise. However, it is important to notice that the type of population and the type of exercise differed between the current study and the previous research. Primarily, the current study included participants free from diabetes, where the relationship between FGM‐derived glucose fluctuations and health outcomes is not as well established (Guess, 2023).

The current study also investigated whether the time of day at which resistance exercise is performed influences adaptations in muscle strength and size. Previous research found that maximum strength peaks late afternoon with exercise compared to morning exercise (Drust et al., 2005), and this is often cited as a reason to hypothesize that muscular adaptations to resistance exercise will be superior in the afternoon/evening. Analysis of training volume data revealed no difference in exercise loads between morning and evening groups across all exercises performed. Furthermore, the current data found no clear effect of time of day on the adaptations to resistance exercise training, although these were relatively modest given the short 6‐week duration of the current intervention. This is in agreement with a previous systematic review and meta‐analysis on the topic (Grgic et al., 2019). In this review, it was shown, based on 11 studies, that increases in knee extensor maximal torque do not differ when comparing resistance training in the morning versus the evening (*P* = 0.220; standardised mean diffeerence (SMD) = 0.19, 95% CI = −0.11, 0.50; *I* ^2^ = 0%). Similar results were found for muscle mass, where, based on five studies, increases in muscle mass were similar when comparing resistance exercise training in the morning versus the evening (SMD = 0.20, 95% CI: −0.40, 0.40; *P* = 0.958; *I* ^2^ = 0%). The current study, therefore, supports these findings, and, in our opinion, it is clear that there is no effect of the timing of resistance exercise on adaptations in muscle strength and mass.

The current study is not without limitations, and it is prudent to consider these, alongside its strengths. A major strength of this study is that it followed a randomised controlled trial design. We employed blinded FGMs to ensure that seeing these data did not result in behavioural changes. We use the real‐time FGM (rtFGM) due to the available technology. Moreover, the strict timing and the structure of the exercise sessions ensure study validity. A primary limitation is the study sample characteristics, particularly being healthy young adults, which limits the generalisability to all other populations. An important limitation of this study is that no formal a priori power analysis was conducted. Furthermore, the study duration is 6 weeks, which means adaptations to resistance exercise were relatively small, particularly for muscle strength and size, and so the potential for these to be influenced by the time of day was relatively small. A further limitation of the current study is that dietary intake was not systematically controlled, which may have influenced the result. An additional limitation is that participants were randomly assigned to the intervention without consideration of their individual chronotype. Previous studies have shown that morning exercise has the greatest potential to alleviate circadian misalignment in young adults (Thomas et al., 2020). This approach may mean that some participants were exercising at times misaligned with their natural circadian preferences. Future studies should consider chronotype‐matched exercise timing to better understand circadian influences on exercise adoption. Moreover, the current study did not use the gold standard measure of muscle mass, although the use of ultrasound has been validated (Franchi et al., 2018). Insulin sensitivity was measured during an OGTT rather than the golden standard hyperinsulinaemic euglycaemic clamp.

This study demonstrated that 6 weeks of resistance exercise resulted in improvements in muscle strength, muscle mass and insulin sensitivity in healthy young adults, regardless of whether training occurred in the morning or evening. The lack of time‐of‐day effects was consistent across all measured outcomes, including FGM‐derived glucose metrics, muscle strength and body composition measures. With the lack of effect of the time of day at which exercise is performed, public health strategies should promote resistance exercise at times convenient to the individual to ensure its benefits.

## AUTHOR CONTRIBUTIONS

Drafted the manuscript and performed statistical analysis: Anas Dighriri. Conceived and designed the study: Stuart R. Gray and Anas Dighriri. Contributed to the study design: Greig Logan and Brendan Gabriel. Contributed to data collection: Emma Dunning, Lynsey Johnston, Hannah Lithgow, Anas Dighriri and Mazin Altuwrqi. Critically revised the manuscript: Stuart R. Gray, James G Boyle, Lynsey Johnston, Emma Dunning, Brendan Gabriel, Hannah Lithgow and Greig Logan. Recruited participants: Anas Dighriri and Mazin Altuwrqi. Supervised all aspects of the research, provided critical expertise throughout: Stuart R. Gray. Provided expertise in metabolic health: James G Boyle. Supervised exercise interventions: Greig Logan. Provided expertise in exercise and circadian rhythm: Brendan Gabriel. All authors approved the final version of the manuscript, agree to be accountable for all aspects of the work, and confirm that all persons designated as authors qualify for authorship and all those who qualify for authorship are listed. All experiments were performed at the School of Cardiovascular & Metabolic Health, University of Glasgow, Glasgow, United Kingdom.

## CONFLICT OF INTEREST

The authors declare no conflicts of interest relevant to this study.

## FUNDING INFORMATION

No funding was received for this work.

## Data Availability

The datasets generated and analysed during the current study are available from the corresponding author on reasonable request. Individual participant data cannot be publicly shared due to privacy and ethical restrictions, but aggregate data supporting the conclusions are contained within the manuscript and its tables.
